# DNA Barcoding of Shark Meats Identify Species Composition and CITES-Listed Species from the Markets in Taiwan

**DOI:** 10.1371/journal.pone.0079373

**Published:** 2013-11-18

**Authors:** Shang-Yin Vanson Liu, Chia-Ling Carynn Chan, Oceana Lin, Chieh-Shen Hu, Chaolun Allen Chen

**Affiliations:** 1 Biodiversity Research Center, Academia Sinica, Nangang, Taipei, Taiwan; 2 Department of Ecology and Evolutionary Biology, University of California Los Angeles, Los Angeles, United States of America; 3 The Society of Wilderness, Taipei, Taiwan; 4 Taiwan International Graduate Program-Biodiversity, Academia Sinica, Nangang, Taipei, Taiwan; 5 Institute of Oceanography, National Taiwan University, Taipei, Taiwan; Auburn University, United States of America

## Abstract

**Background:**

An increasing awareness of the vulnerability of sharks to exploitation by shark finning has contributed to a growing concern about an unsustainable shark fishery. Taiwan’s fleet has the 4th largest shark catch in the world, accounting for almost 6% of the global figures. Revealing the diversity of sharks consumed by Taiwanese is important in designing conservation plans. However, fins make up less than 5% of the total body weight of a shark, and their bodies are sold as filets in the market, making it difficult or impossible to identify species using morphological traits.

**Methods:**

In the present study, we adopted a DNA barcoding technique using a 391-bp fragment of the mitochondrial cytochrome oxidase I (COI) gene to examine the diversity of shark filets and fins collected from markets and restaurants island-wide in Taiwan.

**Results:**

Amongst the 548 tissue samples collected and sequenced, 20 major clusters were apparent by phylogenetic analyses, each of them containing individuals belonging to the same species (most with more than 95% bootstrap values), corresponding to 20 species of sharks. Additionally, *Alopias pelagicus, Carcharhinus falciformis*, *Isurus oxyrinchus,* and *Prionace glauca* consisted of 80% of the samples we collected, indicating that these species might be heavily consumed in Taiwan. Approximately 5% of the tissue samples used in this study were identified as species listed in CITES Appendix II, including two species of *Sphyrna*, *C. longimanus* and *Carcharodon carcharias*.

**Conclusion:**

DNA barcoding provides an alternative method for understanding shark species composition when species-specific data is unavailable. Considering the global population decline, stock assessments of Appendix II species and highly consumed species are needed to accomplish the ultimate goal of shark conservation.

## Introduction

Unsustainable fishing pressure has led to the decline of most shark populations, and some are facing extinction [1], [2], [3]. These predators play a crucial ecological role in structuring marine ecosystems and food webs [4], and are commercially important for their meat and particularly their fins. Late maturation, low fecundity, and longevity make sharks acutely vulnerable to overexploitation and prevent rapid recovery from over-fishing [5].

Recent global catch assessments estimated approximately 100 million sharks are landed annually, excluding illegal, unreported, and unregulated shark catches [3]. Evidence of continuing over-fishing of shark populations triggered immediate conservation actions by the Food and Agriculture Organization of the United Nations (FAO), international treaties such as the Convention on International Trade in Endangered Species of Wild Fauna and Flora (CITES), and the creation of regional fisheries management organizations (RFMOs) by shark harvesting countries and entities. A review of global actions and inaction on sharks [6] reported the global shark fishery is primarily driven by 20 countries, with Indonesia (13%), India (9%), Spain (7.3%), Taiwan (5.8%), and Argentina (4.3%) contributing most to shark landings. Thirteen shark harvesting countries are known to have national plans of action for conserving and managing sharks (NPOA-Sharks). However, no substantial evidence exists to indicate that NPOAs are increasing the effective management of shark fisheries in their countries [6].

Taiwan’s fleet has the 4th largest shark catch in the world, with a declared 6 million sharks caught annually, accounting for almost 6% of the global figures. However, these numbers could be greatly underestimated. Biogeographically, Taiwan has the highest species diversity of sharks in the world [7]. Between 1996 and 2006, annual Taiwanese shark landings (coastal, offshore, and pelagic combined) averaged between 39,000 and 55,000 metric tons. Sharks are captured primarily by bottom longline, mid-water longline, large-mesh drift-net, and as by-catch of the tuna longline fishery. The dominant species are *Prionace glauca* (blue shark), *Isurus oxyrinchus* (shortfin mako shark), *Sphyrna lewini* (scalloped hammerhead shark), *S. zygaena* (smooth hammerhead shark), *Alopias superciliosus* (bigeye thresher shark), *A. pelagicus* (pelagic thresher shark), *Carcharhinus plumbeus* (sandbar shark), *C. falciformi*s (silky shark), *C. longimanus* (oceanic whitetip shark), *C. brevipinna* (spinner shark), and *C. obscurus* (dusky shark) [8].

The Taiwanese government initiated NPOA-Sharks on 05 May 2006 [8], and Taiwan became the first Asian nation to implement a ban on shark finning in early 2012. The new law requires that sharks be landed naturally with their fins attached, where fishes are to be inspected and identified, and then processed at port. Fleets that violate the regulations are heavily fined and may have their fishing licenses revoked.

Stock assessments have been severely hampered by the lack of species-specific catch data in most fisheries, especially sharks [9]. The shark fisheries data released by the Fisheries Agency, Council of Agriculture, Taiwan (FA-COA) contain no species-specific catch data, which is mainly due to unintegrated catch data from landings, commercial fishing vessels, and sampling vessels [8]. Furthermore, the shark catch data in the Taiwan fisheries yearbook contains only five generic categories classifying landings by shape and size, with no regard to species identification. This phenomenon is common in countries with shark fishing activities. Only 6 of the top 20 countries that land sharks provide species names to the FAO [6].

Species-level identification of harvested individuals is critical for the development of protocols for sustainable fisheries management. However, field identification of some closely related shark species, such as carcharhinid sharks [10], is difficult. Additionally, shark fins and meats in the market are highly processed, preventing morphological identification and thus requiring additional methods to identify captured sharks to species. DNA barcoding using COI is a powerful tool for fish species identification [11], [12], [13], [14], and several studies have successfully used COI to barcode sharks [15], [16], [17], [18], [19]. Currently, FISH-BOL has recorded 53% of the 1178 recognized shark and ray species in the database as of 2013 (http://www.fishbol.org/progress_reports.php?region=1&lvl=genus&type=Elasmobranchii), providing an excellent resource of genetic data that can be utilized for Taiwanese shark management efforts.

Here we characterize a molecular method to identify shark meat from the local markets of Taiwan using genetic barcoding. Our efforts reveal shark species composition in Taiwanese markets and support the utility of DNA barcoding for species identification and classification by IUCN population status. This will provide the necessary species-specific data to the authorities responsible for managing shark stocks.

## Materials and Methods

**Figure 1:**
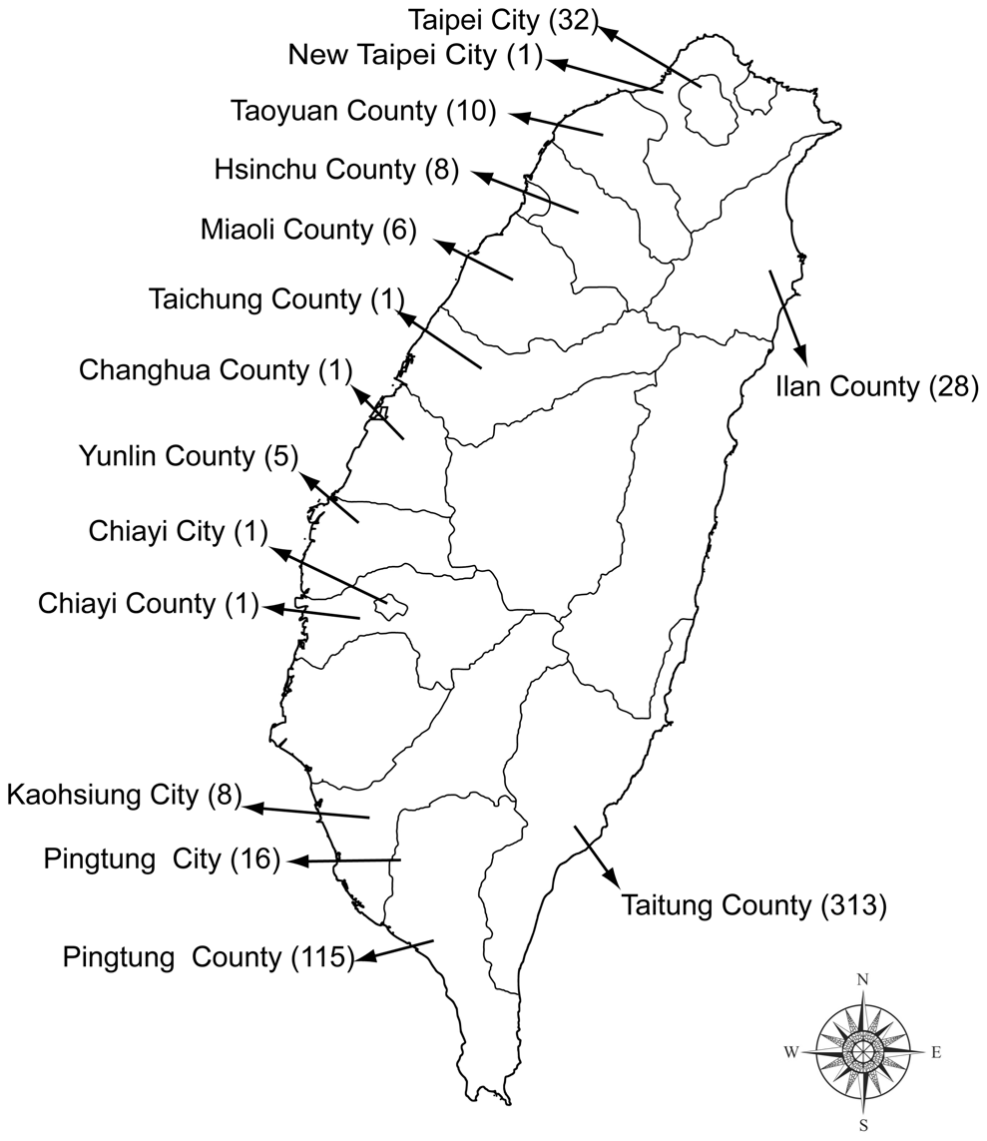
Map of Taiwan showing sampling areas for shark tissues used in this study. Numbers in parentheses indicate sample sizes, and solid lines indicate county borders.

Shark tissues were collected from June 2011 to January 2013 from harbor loadings, fish markets, supermarkets, street venders, and restaurants in six cities and eight counties of Taiwan (Fig. 1) with the help of volunteers from the Society of Wilderness. Considering that an individual shark can be cut into many fillets, we avoided purchasing multiple tissue samples from a single source and collected only one tissue sample from one individual from the fresh landing to reduce possible sampling redundancy. A total of 548 samples was purchased and preserved in 95% ETOH for DNA extraction.

DNA was isolated with the Genomic DNA extraction kit (Genomics BioSci. and Tech. Co., Taiwan) from muscle tissue according to the manufacturer’s recommendations. A partial fragment of the mitochondrial (mtDNA) COI gene was amplified by polymerase chain reaction (PCR) using Taq DNA polymerase (MDbio, Taipei) with universal primers LCO1490∶5′-GGTCAACAAATCATAAAGATA TTGG-3′ and HCO2198∶5′-TAAACTTCAGGGTGACCAAAAAATCA-3′ [20]. Each 25 µl reaction contained 10–50 ng DNA, 10 mM Tris HCl (pH 8.3), 50 mM KCl, 1.5 mM MgCl2, 1 U Taq DNA polymerase, 0.2 mM dNTPs, and 0.3 mM of each primer. The mixture was amplified with a cycling profile of 2 min at 94°C, followed by 34 cycles at 95°C (30 s), 54°C (30 s), and 70°C (40 s). The nucleotide sequences of the PCR products were determined using an ABI 377 automated sequencer with the forward and reverse primers used for amplification. Sequences were aligned using CLUSTAL W [21] and followed by manual editing using Sequencher 4.2 (Gene Code, Ann Arbor, MI, USA). Sequences used in this study were submitted to the NCBI GenBank database (accession: KF606764–KF606860). Obtained sequences were blasted through NCBI and BOLD web-based systems. The hits with the highest query coverage and maximum identical values (>98%) were chosen as reference sequences. These sequences, as well as 44 more (representing 22 species) downloaded from GenBank, including 31 voucher and 13 non-voucher samples (Table S1), were added to the aligned sequences generated in this study for further phylogenetic analyses.

Phylogenetic analyses were performed using neighbor-joining (NJ) and maximum-likelihood (ML) methods. The best-fitting substitution model, HKY+Gamma (Gamma = 0.1869), was selected by MEGA 5 [22] and applied to neighbor- joining tree construction in MEGA 5. In addition, RAxML-HPC [23] was used to perform phylogenetic analysis using maximum-likelihood methods. To evaluate the robustness of the internal branches of the ML tree, 500 bootstrap replications (BS) were run under the GTR+ Gamma model. Sequence divergences between species were calculated by MEGA 5 using the Kimura two parameter (K2P) distance model [24].

## Results

All samples were successfully amplified, however, some of reads from reversed direction had difficulty to read (5′ region). Therefore, a trimmed sequence of 391 base-pairs was obtained for the partial COI region in 548 shark samples. Of these, 155 sites were variable and 141 were parsimony-informative sites. Mean nucleotide composition was 25.5% thymine, 26.1% cytosine, 33.1% adenine, and 26.6% guanine.

**Figure 2:**
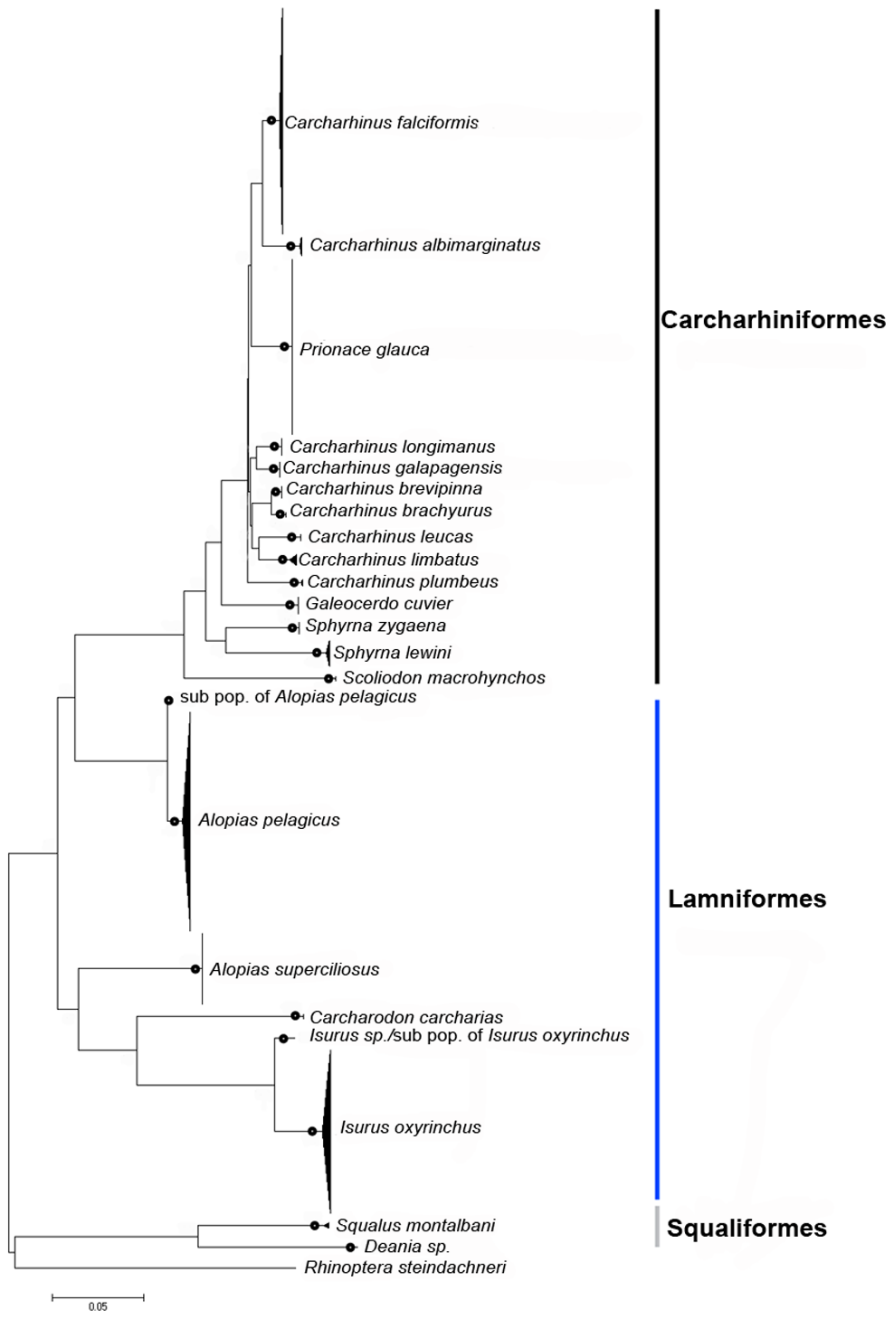
Neighbor-joining tree of 548 COI gene sequences with 44 sequences downloaded from GenBank, corresponding to 20 shark species with *Rhinoptera steindacheri* as the outgroup. The black dot indicates a bootstrap value >95%.

The neighbor-joining tree supported 20 species-specific clades with >95% bootstrap support (Fig. 2). These 20 species-specific clades spanned three orders of sharks: the Carcharhiniformes, Lamiformes and Squaliformes. Within the Carcharhiniformes, nine monophyletic species-specific clades of *Carcharhinus* were recovered: *C. albimarginatus* (silvertip shark), *C. plumbeus*, *C. brachyurus* (copper shark), *C. brevipinna*, *C. falciformis*, *C. galapagensis* (Galapagos shark), *C. leucas* (bull shark), *C. limbatus* (blacktip shark), and *C. longimanus*. Clades of *Prionace glauca*, *Scoliodon laticaudus* (spadenose shark), *Galeocerdo cuvier* (tiger shark), and two species of *Sphyrna* (*S. lewini* and *S. zygaena*) were also recovered with strong bootstrap support. *Carcharodon carcharias* (white shark), *Isurus oxyrinchus*, and two species of *Alopias, A. pelagicus* and *A. superciliosus*, belong to the Laminiformes. *Squalus montalbani* (Philippines spurdog) and *Deania* sp. (dogfish shark) belong to the Squaliformes. According to BLAST results, *Deania sp.* is most closely related to *D. calcea* (DQ108224.1), but with only 95% sequence similarity. However, the result of the BOLD engine search revealed that *D. quadrispinosa* was most closely related to our *Deania* sample, but sequence data has not yet been released to the public. Therefore, this species will be considered as *Deania* sp. in this study.

Three non-voucher samples, including *Carcharodon carcharias*, *Lamna nasus,* and *Isurus oxyrinchus* (Table S1) clustered with three voucher species, *Prionace glauca*, *Carcharhinus galapagensis,* and *Carcharhinus falciformis,* respectively. With further deployment of the BOLD search engine, results confirmed that these non-voucher sequences are case of misidentification. ‘In addition, a genetically distinct sub-population or cryptic species was found within each of the *A. pelagicus* and *I. oxyrinchus* lineages (Fig. 2).

**Figure 3:**
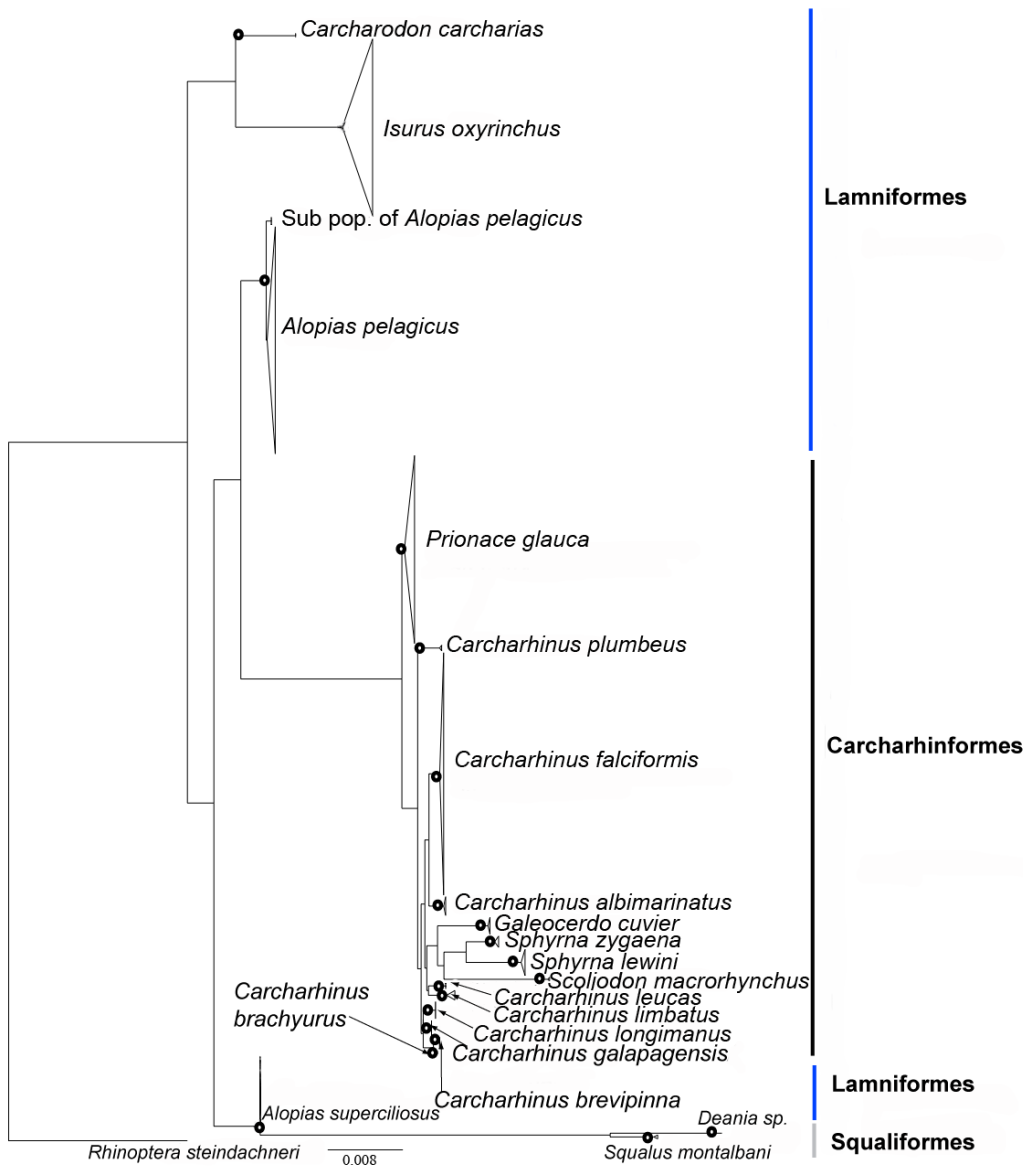
Maximum-likelihood tree of 548 COI gene sequences with 44 sequences downloaded from GenBank, corresponding to 20 shark species with *Rhinoptera steindacheri* as the outgroup. The black dot indicates a bootstrap value >95%.

In general, the ML tree showed a different tree topology from the NJ tree. The Lamniformes did not form a monophyletic group and *A. superciliosus* grouped with Squaliformes. Additionally, the bootstrap values for basal branches were lower than 50%. Furthermore, the derived branch of *I. oxyrinchus* was not recovered in the ML tree (Fig. 3).

According to the detailed collection information for the 548 samples, 57% of samples were collected from Taitung County and 21% from Pingtung County. In addition, the identification of sharks as revealed by genetic barcoding was used to check the species name corresponding to where it was collected (Table S2). *Alopias pelagicus* (N = 125), *Carcharhinus falciformis* (N = 125), *Isurus oxyrinchus* (N = 92), and *Prionace glauca* (N = 98) represented 80% of shark meats that we collected in Taiwan. Five percent of the samples used in this study were identified as species listed in CITES Appendix II, including *Carcharodon carcharias*, *Carcharhinus longimanus*, *Sphyrna lewini,* and *S. zygaena*. Following IUCN classifications (EN: endangered, VU: vulnerable, NT: near threatened, and LC: least concern), 2.5% of the samples were classified as EN, 50% as VU, 24.5% as NT, and 23% as LS (Table 1). These values excluded the *Deania sp.* that could not be identified to species. The K2P genetic distance between species ranged 0.013–0.308, within genus ranged 0.042–0.109 (only three genera had sample sizes greater than two individuals, including *Alopias*, *Carcharhinus* and *Sphyrna*), and between order ranged 0.2–0.29 (Carcharhiniformes, Lamniformes, and Squaliformes) (Table 2).

**Table 1:** A list of 20 species identified by genetic barcoding.

Species name	Abb.	IUCN	CITES Appendix II	N	Averaged Landing %
*Alopias pelagicus*	Apel	VU		125	3.66
*Alopias superciliosus*	Alop	VU		39	5.18
*Carcharhinus albimarginatus*	Calb	NT		9	
*Carcharhinus plumbeus*	Cplu	VU		1	2.25
*Carcharhinus brachyurus*	Cbra	NT		1	
*Carcharhinus brevipinna*	Cbre	NT		5	2.34
*Carcharhinus falciformis*	Cfal	NT		125	1.04
*Carcharhinus galapagensis*	Cgal	NT		5	
*Carcharhinus leucas*	Cleu	NT		2	
*Carcharhinus limbatus*	Clim	NT		4	
*Carcharhinus longimanus*	Clon	VU	V	9	0.38
*Carcharodon carcharias*	Ccar	VU	V	1	
*Galeocerdo cuvier*	Gcuv	NT		8	
*Isurus oxyrinchus*	Ioxy	VU		92	9.42
*Prionace glauca*	Pgla	NT		98	44.54
*Scoliodon laticaudus*	Smac	NT		2	
*Sphyrna lewini*	Slew	EN	V	14	9.87
*Sphyrna zygaena*	Szyg	VU	V	5	3.66
*Deania sp.*	Dsp			1	
*Squalus montalbani*	Smon	VU		2	
Total				548	

IUCN: population status; CITES Appendix II, population status; N: number of samples identified as the indicated shark species; Abb.: abbreviation of species name. Averaged Landing data is extracted from [44].

**Table 2:** K2P distances among 20 shark species identified by genetic barcoding; species name abbreviations are listed in Table 1.

	Cbra	Pgla	Cbre	Cgal	Cleu	Clim	Clon	Cplu	Calb	Cfal	Gcuv	Szyg	Slew	Smac	Asup	Apel	Ccar	Ioxy	Dsp	Smon
**Cbra**	_																			
**Pgla**	0.050	_																		
**Cbre**	0.013	0.045	_																	
**Cgal**	0.030	0.039	0.033	_																
**Cleu**	0.039	0.062	0.047	0.042	_															
**Clim**	0.044	0.056	0.044	0.050	0.044	_														
**Clon**	0.036	0.042	0.039	0.027	0.047	0.039	_													
**Cplu**	0.041	0.050	0.050	0.047	0.053	0.059	0.053	_												
**Calb**	0.037	0.055	0.048	0.046	0.048	0.051	0.046	0.057	_											
**Cfal**	0.038	0.039	0.038	0.036	0.044	0.044	0.036	0.047	0.031	_										
**Gcuv**	0.089	0.076	0.080	0.074	0.089	0.080	0.071	0.086	0.082	0.073	_									
**Szyg**	0.095	0.104	0.092	0.082	0.085	0.088	0.089	0.107	0.094	0.082	0.105	_								
**Slew**	0.112	0.112	0.106	0.106	0.109	0.113	0.112	0.121	0.096	0.106	0.125	0.097	_							
**Smac**	0.132	0.138	0.125	0.118	0.125	0.135	0.135	0.128	0.131	0.134	0.142	0.155	0.159	_						
**Asup**	0.188	0.174	0.181	0.195	0.181	0.183	0.199	0.184	0.192	0.191	0.192	0.187	0.188	0.216	_					
**Apel**	0.167	0.151	0.160	0.167	0.160	0.161	0.167	0.170	0.161	0.171	0.151	0.153	0.189	0.196	0.108	_				
**Ccar**	0.247	0.229	0.247	0.259	0.231	0.253	0.259	0.254	0.236	0.246	0.236	0.223	0.244	0.245	0.164	0.180	_			
**Ioxy**	0.254	0.252	0.262	0.274	0.260	0.258	0.260	0.261	0.245	0.256	0.239	0.277	0.268	0.294	0.203	0.210	0.171	_		
**Dsp**	0.287	0.283	0.300	0.296	0.279	0.278	0.304	0.283	0.303	0.304	0.279	0.292	0.308	0.289	0.265	0.252	0.281	0.298	_	
**Smon**	0.280	0.287	0.284	0.284	0.276	0.272	0.284	0.255	0.279	0.291	0.263	0.281	0.289	0.244	0.268	0.244	0.275	0.311	0.156	_

## Discussion

DNA barcoding using the mtDNA COI gene is a powerful tool for identifying fish species when there is a lack of morphological data. This method has been broadly used for taxonomy, species delimitation, population and phylogeographic analyses, egg and larvae detection, and industrial applications [25], [19], [26], [27]. It could also provide useful information on the phylogenetic relationships of sharks [28]. The non-monophyly of Carcharhiniformes has been observed with a different combination of molecular markers [29], [30], [31], [32]. Additionally, several closely related species of the genus *Carcharhinus* are similar to each other and difficult to identify. For example, *Carcharhinus limbatus* and *C. tilstoni* are two blacktip species that are morphologically indistinguishable [33] except for precaudal vertebral counts (PCV) that separate them [34]. The difficulty in identifying *Carcharhinus leucas*, *C. amboinensis*, *C. tilstoni*, *C. sorrah,* and *C. brevipinna* in Australian waters has also been mentioned [10]. However, the COI region has been successfully applied to identify these closely related species [15], [10].

In the present study, our NJ tree placed *Prionace glauca* within the genus *Carcharhinus*. The *Sphyrna* lineage, *Scoliodon laticaudus,* and *Galeocerdo cuvier* were some of the first lineages to branch off at the base of the Carcharhiniform clade. These results are concordant with previous studies based on ITS1-2 and mtDNA genes [35], [36], [32]. The ML tree, however, showed a different phylogenetic relationship in Carcharhiniformes. The *Sphyrna* lineage and *Galeocerdo cuvier* fell within the genus *Carcharhinus*, and *Prionace glauca* was sister to the *Carcharhinus* clade. Phylogenetic discordance was also observed in the Lamniformes (Figs. 2, 3). According to the morphological and molecular phylogeny of Lamniformes, *Alopias pelagicus* and *A. superciliosus* form a strongly supported monophyletic clade [41]. However, neither the NJ nor ML trees in this study support this relationship. In general, the bootstrap values on the basal branches were lower than 50%, suggesting that the highly variable partial COI gene may not be suitable for resolving higher-level taxonomic phylogenetic relationships. In terms of lower-level phylogenetic relationships, the results of our species-level identifications are robust and accurate. Every sequence used in this study could be matched to a specific species name in the reference database (Fish-BOLD/GenBank), except for one sequence identified to genus level (*Deania sp.*).

Genetic divergence between geographic localities is commonly found in chondrichthyans [15]. Increasing numbers of genetically cryptic elasmobranch species are being identified, such as the scalloped hammerhead *Sphyrna lewini* [37], [38] and wobbegong sharks *Orectolobus* sp. [39]. Intra-specific variation was revealed by this study in two pelagic species, *Isurus oxyrinchus* and *Alopias pelagicus.* A shark genetic barcoding study conducted in 2008 [15] suggested that the unusually high intraspecific genetic distance of 1.2% found in *Isurus oxyrinchus* might reflect high intraspecific diversity rather than cryptic speciation. Therefore, the subclade of *Isurus oxyrinchus* found in this study could be due to high intraspecific diversity as previous studies suggest. On the other hand, previous work suggests a strict genetic break between eastern and western Pacific populations of *Alopias pelagicus* [40]. Our findings recovered two distinct clades of *Alopias pelagicus* in Taiwanese waters, which is consistent with the result found by [41]. This could be due to mixed landings from distant seas, to coastal fleets fishing different populations, or to the sympatric occurrence of both populations in Taiwanese waters. To clarify this issue, detailed species-specific landing data must be incorporated with genetic barcoding for future stock management.

According to the genetic barcoding results, several shark species are heavily consumed by Taiwanese, including *Alopias pelagicus, Carcharhinus falciformis*, *Isurus oxyrinchus*, and *Prionace glauca*. The pelagic thresher, *Alopias pelagicus*, is widely distributed in tropical and subtropical waters [42]. This species has been a common and commercially important shark in Taiwan fisheries since the 1930s [43]. The pelagic thresher composed an average of 11.9% of the Nanfanao fish market’s total landing weight in 1989–2002 [8], but dropped to an average of 3.66% in 2001–2010 [44]. A recent study [45] based on a stochastic stage-based model suggested that the northwestern Pacific pelagic thresher stock has been reduced 34.3% over the past 20 years and that this stock is overexploited. In addition, the global population has been significantly decreasing and the species has been placed in the IUCN red list as vulnerable [46]. Approximately 23% of the tissue samples used in this study is identified as pelagic thresher shark, which could indicate that this species is under high fishing pressure in Taiwan. According to [45], this species is extremely vulnerable to overexploitation and is sensitive at juvenile and adult stages. Therefore, the closure of nursery grounds or the institution of a size limit is urgently needed to ensure the sustainable utilization of the stock. The silky shark (*Carcharhinus falciformis*) is valuable to a wide variety of pelagic fisheries and is taken in large numbers, but there are no population estimates and most catches are unreported. Silky sharks rank among the three most important sharks in the global shark fin trade [47], with 0.5–1.5 million traded annually. In Taiwanese waters, the average annual landing (whole weight) of sharks at Nanfangao was 5,669 MT in 2001–2010. Reported silky shark landings only composed 1.04% of the total shark landings [44]; however, approximately 23% of our tissue samples were from the silky shark. This suggests an increased level of silky shark exploitation in the past few years, a contribution of fish landings from other harbors, or contributions by un-reported landings. However, it is difficult to determine the reason for this discrepancy due to the lack of species-specific landing data. According to [48], the global silky shark population is decreasing and *Carcharhinus falciformis* has therefore been placed on the IUCN red list under the criteria of near threatened and could meet the criteria for VU in the future.

The shortfin mako (*Isurus oxyrinchus*) is an important species for pelagic longline, drifting, and set gill nets, and for hook-and-line fisheries in the eastern Pacific [49]. An instantaneous rate of decline of 38% between 1992 and 2005 in the Northwest Atlantic and Gulf of Mexico has been assessed [50]. This species is taken by tuna and shark longline fisheries in Indonesia [51] and is a common bycatch in tuna and billfish longline and driftnet fisheries. This shark is commonly found in the waters of northeastern Taiwan, and annual production at the Nanfangao fish market (located on the northeast coast of Taiwan) can reach 420 tons [52]. Even though species-specific catch data is not currently available for Taiwan, and given the declines observed where it is heavily fished, we conclude that the stock in Taiwanese waters may also be experiencing high levels of exploitation. According to [48], the global population of the shortfin mako is decreasing and the species has been placed on the IUCN red list under the criteria of VU.

The blue shark is an abundant pelagic shark that is widespread in temperate and tropical waters [53]. It is relatively fast-growing and fecund, and matures in 4–6 years with an average litter size of 35 pups. Blue sharks are rarely a target commercial species, but are a major bycatch of longline and driftnet fisheries in the United States, Europe, Taiwan, and Australia [54], [55]. The blue shark was the dominant shark species between 2001 and 2010, making up 44.54% of sales for two major Taiwanese fish markets (average of 2525 MT of total annual landings) [44]. Several stock assessments have been completed in the Atlantic, Pacific and Indian Oceans [56], [57], [58], [59], [60]. Results indicate that the Atlantic population is relatively stable compared to other oceans. Blue sharks in Taiwan are sold in different channels, making it difficult to collect catch data [44]. Therefore, recording species-specific landing data is an important parameter for blue shark stock assessment in Taiwan. The results of DNA barcoding showed that approximately 23% of collected tissue samples were from blue sharks, indicating that it might be the most dominant shark species consumed in Taiwan. This result fits with the catch report of pelagic sharks in the northwestern Pacific [44]. Continuous high fishing pressure may have a great impact on population demography, even on relatively abundant species such as the blue shark. Stock assessment is urgently needed for this species in Taiwanese waters.

Eight species of sharks are currently included in the CITES Appendix II, *Rhincodon typus*, *Cetorhinus maximus*, *Carcharodon carcharias*, *Carcharhinus longimanus*, *Lamna nasus*, *Sphyrna lewini*, *S. mokarran*, and *S. zygaena*. A catch-report system on rare sharks has been implemented by the Taiwanese government since 2001. The length, weight, sex, time, and fishing gear used must be reported to local government. Due to the rise in public awareness for the conservation of whale sharks (*R. typus*), it is the only species with a restricted ban on fishing and trading in Taiwan. Among our 548 tissue samples, none were identified as whale shark, suggesting that implementation of the ban has been successful. Regardless of global population declines in the basking shark and great white shark, they have only been included in the catch-report species list instead of being banned along with the great white shark. In 2001–2010, smooth and scalloped hammerhead sharks comprised 1.38% (78 MT) and 9.37% (531 MT) of the sales of two major fish markets in Taiwan [44]. The average percentage of *Sphyrna lewini* total landing weights in 1989–2002 were 16.8% (320 MT; Nanfanao fish market only), indicating that the fishing intensity for this species is high. Global captures in 2010 were 336 tons, which is lower than capture data for Taiwan alone. However, only seven countries have reported *Sphyrna lewini* data to the FAO, which is by no means an accurate representation of worldwide *S. lewini* landings data. Therefore, the global capture production released by the FAO most likely underestimates global capture. For *Sphyrna zygaena*, captures were much lower than *S. lewini* over the past 20 years [8], [44] in Taiwan, suggesting that its stock could be smaller than *S. lewini* on a regional scale. In the present study, four and 15 samples were identified as smooth and scalloped hammerhead sharks, respectively, composing 3.46% of our samples. This is proportionally lower than expected, but the hammerhead shark fishery undoubtedly has a substantial input to the Taiwanese shark fishery. Since smooth and scalloped hammerhead sharks are both listed by CITES in Appendix II in 2013, actions should be taken for countries such as Taiwan to manage their stocks and decrease the fishing pressure on them.

Although *Cetorhinus maximus* and *Carcharodon carcharias* have been included in the catch-report system, the FA-COA (Taiwan) has not released the data. Only one tissue sample that we collected from Taitung County (Chengkung fish markets) was identified as *Carcharodon carcharias,* and no samples were identified as *Cetorhinus maximus*, suggesting that the capture of these species could be very rare in Taiwanese waters.

Genetic barcoding has been commonly used for shark species identification in certain geographic regions [15], [36], [18], [41]. However, no prior studies have been designed to thoroughly sample and quantify shark meat consumption in real markets. Accordingly, we collected samples for this study across counties and cities of Taiwan where the citizens are able to purchase shark meat. The species list we have assembled from genetic barcoding is similar to the species list derived by using annual landing data. Ten of the 11 dominant species mentioned in NOPA-Shark Taiwan were on our species list, the exception being *Carcharhinus obscurus*. However, Naylor et al. [41] found that *C. obscurus* is genetically similar to *C. galapagensis* for mtDNA ND2 which evolve faster than COI. Therefore, there is a high possibility these two species maybe the one and the same. Further investigation should be proceeded by using highly variable loci such as microsatellite or single-nucleotide polymorphism (SNP) to resolve this species boundary question. Our results show that market species composition might reflect a similar pattern to that of landing data, suggesting that genetic barcoding could be an alternative way to acquire species-specific data from markets. Meanwhile, based on the current population status of sharks on regional and global scales, we suggest that fishing and trading in Appendix II species should be banned and that fishing quotas be gradually decreased for highly consumed species to conserve the wild populations surrounding Taiwan.

## Supporting Information

Table S1 Reference sequences downloaded from GenBank. Columns in gray indicated possible misidentifications.
(DOCX)

Table S2 Results of genetic barcoding and the species composition in different sampling regions.
(DOCX)

## References

[1] Dulvy NK, Baum JK, Clarke S, Compagno LJV, Cortés E, et al (2008) You can swim but you can’t hide: the global status and conservation of oceanic pelagic sharks and rays. Aquat Conserv 18: 459–482.

[2] Ferretti F, Myers RA, Serena F, Lotze HK (2008) Loss of large predatory sharks from the Mediterranean Sea. Conserv Biol 22: 952–964. 18544092 10.1111/j.1523-1739.2008.00938.x

[3] Worm B, Davis B, Kettemer L, AW-P Christine, Chapman D, et al (2013) Global catches, exploitation rates, and rebuilding options for sharks. Mar Policy 40: 194–204.

[4] Libralato S, Christensen V, Pauly D (2005) A method for identifying keystone species in food web models. Ecol Model 195: 153–171.

[5] Stevens JD (1999) Variable resistance to fishing pressure in two sharks: The significance of different ecological and life history parameters. In: editor Musick JA Life in the Slow Lane: Ecology and Conservation of Long-Lived Marine Animals American Fisheries Society, Bethesda, MD, 11–15.

[6] Lack M, Sant G (2011). The Future of Sharks: A Review of Action and Inaction. TRAFFIC International and the Pew Environment Group.

[7] Lucifora LO, Garcı’a VB, Worm B (2011) Global Diversity Hotspots and Conservation Priorities for Sharks. PLoS ONE 6: e19356. 21573162 10.1371/journal.pone.0019356 PMC3088674

[8] Taiwan Fisheries Agency (2006) Taiwan’s National Plan of Action for the Conservation and Management of Sharks. Fisheries Agency, Taipei, Taiwan. www.fa.gov.tw/eng/guide/npoasharke.php, accessed 28 January 2007.

[9] Camhi MD (2008) Conservation Status of Pelagic Elasmobranchs. In: Camhi, EK Pikitch, EA Babcock editors Sharks of the open ocean: Biology, Fisheries and Conservation. 397–417.

[10] Tillett BJ, Field IC, Johnson G, Buckworth R, Meekan MG, et al (2012) Accuracy of species identification by fisheries observers in a north Australian shark fishery. Fish Res 127–128: 109–115.

[11] Ward RD, Zemlak TS, Innes BH, Last PR, Hebert PDN (2005) DNA barcoding Australia’s fish species. Philos Trans R Soc Lond B Biol Sci 360: 1847–1857. 16214743 10.1098/rstb.2005.1716 PMC1609232

[12] Hubert N, Hanner R, Holm E, Mandrak NE, Taylor E, et al (2008) Identifying Canadian Freshwater Fishes through DNA Barcodes. PLoS ONE 3: e2490. 22423312 10.1371/journal.pone.0002490 PMC3278308

[13] Steinke D, Zemlak TS, Hebert PDN (2009) Barcoding Nemo: DNA-based identifications for the ornamental fish trade. PLoS ONE 4: e6300. 19621079 10.1371/journal.pone.0006300 PMC2708913

[14] Ward RD, Hanner R, Hebert PDN (2009) The campaign to DNA barcode all fishes, FISH-BOL. J Fish Biol 74: 329–356. 20735564 10.1111/j.1095-8649.2008.02080.x

[15] Ward RD, Holmes BH, White WT, Last PR (2008) DNA barcoding Australasian chondrichthyans: results and possible uses in conservation. Mar Freshwater Res 59: 57–71.

[16] Holmes BH, Steinke D, Ward RD (2009) Identification of shark and ray fins using DNA barcoding. Fish Res 95: 280–288.

[17] Wong EH-K, Shivji MS, Hanner RH (2009) Identifying sharks with DNA barcodes: assessing the utility of a nucleotide diagnostic approach. Mol Ecol Resour 9 (s1): 243–256. 10.1111/j.1755-0998.2009.02653.x 21564984

[18] Moftah M, Abdel Aziz SH, Elramah S, Favereaux A (2011) Classification of Sharks in the Egyptian Mediterranean Waters Using Morphological and DNA Barcoding Approaches. PLoS ONE 6: e27001. 22087242 10.1371/journal.pone.0027001 PMC3206905

[19] Ward RD (2009) Shark fin identification through DNA barcoding. Endangered species update 26: 3–9.

[20] Folmer O, Black M, Hoeh W, Lutz R, Vrijenhoek R (1994) DNA primers for amplification of mitochondrial cytochrome c oxidase subunit I from diverse metazoan invertebrates. Mol Mar Biol Biotechnol 3: 294–297. 7881515

[21] Thompson JD, Higgins DG, Gibson TJ (1994) CLUSTAL W: improving the sensitivity of progressive multiple sequence alignment through sequence weighting, position-specific gap penalties and weight matrix choice. Nucleic Acids Res 22: 4673–4680. 7984417 10.1093/nar/22.22.4673 PMC308517

[22] Tamura K, Peterson D, Peterson N, Stecher G, Nei M, et al (2011) MEGA5: Molecular Evolutionary Genetics Analysis using Maximum Likelihood, Evolutionary Distance, and Maximum Parsimony Methods. Mol Biol Evol 28: 2731–2739. 21546353 10.1093/molbev/msr121 PMC3203626

[23] Stamatakis A (2006) RAxML-VI-HPC: maximum likelihood-based phylogenetic analyses with thousands oftaxa and mixed models. Bioinformatics 22: 2688–2690. 16928733 10.1093/bioinformatics/btl446

[24] Kimura M (1980) A simple model for estimating evolutionary rates of base substitutions through comparative studies of nucleotide sequences. J Mol Evol 16: 111–120. 7463489 10.1007/BF01731581

[25] Clarke SC, Magnussen JE, Abercrombie DL, McAllister MK, Shivji MS (2006) Identification of shark species composition and proportion in the Hong Kong shark fin market based on molecular genetics and trade records. Conserv Biol 20: 201–211. 16909673 10.1111/j.1523-1739.2005.00247.x

[26] Zhang J, Hanner R (2012) Molecular approach to the identification of fish in the South China Sea. PLoS ONE 7(2): e30621. 22363454 10.1371/journal.pone.0030621 PMC3281855

[27] Liu SYV, Ho HHC, Dai CF (2013) A New species of Pomacentrus (Actinopterygii: Pomacentridae) from Micronesia, with comments on its phylogenetic relationships. Zool Stud 52(6).

[28] Martin A, PhD Dissertation, University of Hawaii, Honolulu, HI, 1992.

[29] Iglésias SP, Lecointre G, Sellos DY (2005) Extensive paraphylies within sharks of the order Carcharhiniformes inferred from nuclear and mitochondrial genes. Mol Phylogenet Evol 34: 569–583. 15683930 10.1016/j.ympev.2004.10.022

[30] Human BA, Owen EP, Compagno LJV, Harley EH (2006) Testing morphologically based phylogenetic theories within the cartilaginous fishes with molecular data, with special reference to the catshark family (Chondrichthyes; Scyliorhinidae) and the interrelationships within them. Mol Phylogenet Evol 39: 384–391. 16293425 10.1016/j.ympev.2005.09.009

[31] Lopez JA, Ryburn JA, Fedrigo O, Naylor GJ (2006) Phylogeny of sharks of the family Triakidae (Carcharhiniformes) and its implications for the evolution of carcharhiniform placental viviparity. Mol Phylogenet Evol 40: 50–60. 16564708 10.1016/j.ympev.2006.02.011

[32] Vélez-Zuazo X, Agnarsson I (2011) Shark tales: A molecular species-level phylogeny of sharks (Selachimorpha, Chondrichthyes) Mol Phylogenet Evol. 58: 207–217. 10.1016/j.ympev.2010.11.018 21129490

[33] Compagno LJV, Dando M, Fowler S (2005) Sharks of the World. Princeton University Press, Princeton and Oxford.

[34] Last PR, Stevens JD (2009) Sharks and Rays of Australia. CSIRO Australia, Collingwood, Vic.

[35] Dosay-Akbulut M (2008) The phylogenetic relationship within the genus Carcharhinus. C. R. Biologies 331: 500–509. 18558373 10.1016/j.crvi.2008.04.001

[36] Rodrigues-Filho L, Rocha T, Rêgo P, Schneider H, Sampaio I, et al (2009) Identification and phylogenetic inferences on stocks of sharks affected by the fishing industry off the Northern coast of Brazil. Genet and Mol Biol 32: 405–413. 21637699 10.1590/S1415-47572009005000039 PMC3036939

[37] Duncan KM, Martin AP, Bowen BW, De Couet HG (2006) Global phylogeography of the scalloped hammerhead shark (*Sphyrna lewini*). Mol Ecol 15: 2239–2251. 16780437 10.1111/j.1365-294X.2006.02933.x

[38] Quattro JM, Stoner DS, Driggers WB, Anderson CA, Priede KA, et al (2006) Genetic evidence of cryptic speciation within hammerhead sharks (Genus Sphyrna). Mar Biol 148: 1143–1155.

[39] Corrigan S, Huveneers C, Schwartz TS, Harcourt RG, Beheregaray LB (2008) Genetic and reproductive evidence for two species of ornate wobbegong shark on the Australian East Coast. J Fish Biol 73: 1662–1675.

[40] Trejo T (2005) Global phylogeography of thresher sharks (*Alopias* spp.) inferred from mitochondrial DNA control region sequences”. M.Sc. thesis. Moss Landing Marine Laboratories, California State University.

[41] Naylor GJP, Caira JN, Jensen K, Rosana KAM, White WT, et al (2012) A DNA sequence–based approach to the identification of shark and ray species and its implications for global Elasmobranch diversity and parasitology. Bull Am Mus Nat Hist 367: 1–262.

[42] Compagno LJV (2001) Sharks of the World. Bullhead, mackeral and carpet sharks (Heterodontiformes, Lamniformes and Orectolobiformes). FAO Species Catalogue 2, 78.

[43] Chen GCT, Liu KM, Joung SJ, Phipps MJ (1996) “TRAFFIC report on shark fisheries and trade in Taiwan”, in “TRAFFIC report on shark fisheries and trade in the East Asian region”, of the “The world trade in sharks: a compendium of TRAFFIC’s regional studies volume I”, TRAFFIC.

[44] Liu K-M, Tsai W-P (2011) Catch and life history parameters of pelagic sharks in the Northwest Pacific. ISC Shark Working Group Meeting, April 19–21, Keelung, Taiwan. ISC/11SHARKWG-1/6, 12 pp.

[45] Tsai W-P, Liu K-M, Joung S-J (2010) Demographic analysis of the pelagic thresher shark, *Alopias pelagicus*, in the north-western Pacific using a stochastic stage-based model. Mar Freshwater Res 61: 1056–1066.

[46] Reardon M, Márquez F, Trejo T, Clarke SC (2009) *Alopias pelagicus*. In: IUCN 2012. IUCN Red List of Threatened Species.

[47] Compagno LJV (1984) Sharks of the World. An Annotated and Illustrated Catalogue of Shark Species Known to Date. FAO Species Catalogue. Vol. 4, Part 2. FAO Fisheries Synopsis, 125. FAO, Rome, Italy.

[48] Cailliet GM, Cavanagh RD, Kulka DW, Stevens JD, Soldo A, et al. (2009) *Isurus oxyrinchus*. In: IUCN 2012. IUCN Red List of Threatened Species.

[49] Holts DB, Julian A, Sosa-Nishizaki O, Bartoo NW (1998) Pelagic shark fisheries along the west coast of the United Status and Baja California, Mexico Fish Res. 39: 115–125.

[50] Baum JK, Kehler D, Myers RA (2005) Robust estimates of decline for pelagic shark populations in the Northwest Atlantic and Gulf of Mexico. Fisheries 30: 27–30.

[51] White WT, Last PR, JD Stevens, Yearsley GK, Fahmi, etal. (2006) Economically important sharks and rays of Indonesia. Canberra, Australia, Australian Centre for International Agricultural Research.

[52] Joung SJ, Hsu HH (2005) Reproduction and Embryonic Development of the Shortfin Mako, *Isurus oxyrinchus* Rafinesque, 1810, in the Northwestern Pacific. Zool Stud 44: 337–346.

[53] Campana SE, Marks L, Joyce W, Kohler N (2004) Influence of recreational and commercial fishing on the blue shark (*Prionace glauca*) population in Atlantic Canadian waters. CSAS Res. Doc. 2004/069. 68 p.

[54] Fowler GM, Campana SE (2009) Commercial by-catch rates of blue shark (Prionace glauca) from longline fisheries in the Canadian Atlantic. Collect. Vol. Sci. Pap. ICCAT 64: 1650–1667.

[55] Liu K-M, Joung SJ, Tsai W-P (2009) Preliminary estimates of blue and mako sharks by-catch and CPUE of the Taiwanese longline fishery in the Atlantic Ocean. Collect Vol Sci Pap ICCAT 64: 1703–1716.

[56] Simpfendorfer CA, Heuter RE, Bergman U, Connett SMH (2002) Results of a fishery independent survey for pelagic sharks in the western North Atlantic, 1977–1994. Fisher Res 55: 175–192.

[57] Ward P, Myers RA (2005) Shifts in open-ocean fish communities coinciding with the commencement of commercial fishing. Ecology 86: 835–847.

[58] Nakano H, Clarke S (2005) Standardized CPUE for blue sharks caught by the Japanese longline fishery in the Atlantic Ocean, 1971–2003. ICCAT Collective Volume of Scientific Papers 58: 1127–1134.

[59] Babcock EA, Nakano H (2008) Data collection, research, and assessment efforts for pelagic sharks by the International Commission for the Conservation of Atlantic Tunas. In: Camhi MD, Pikitch EK, Babcock EA editors Sharks of the Open Ocean: Biology, Fisheries and Conservation.Blackwell Publishing, Oxford, UK.

[60] Hueter RE, Simpfendorfer CA (2008) Case study: Trends in blue shark abundance in the western North Atlantic as determined by a fishery-independent survey. In: Camhi MD, Pikitch EK, Babcock EA editors Sharks of the Open Ocean: Biology, Fisheries and Conservation.Blackwell Publishing, Oxford, UK.

