# Supplementary material for: DNA Barcoding of Shark Meats Identify Species Composition and CITES-Listed Species from the Markets in Taiwan
Source: PLoS One. 2013 Nov 18;8(11):e79373. doi: 10.1371/journal.pone.0079373 (PMC3832526; doi:10.1371/journal.pone.0079373)
Supplement: Table S1 — Reference sequences downloaded from GenBank. Columns in gray indicated possible misidentifications. (DOCX) [file pone.0079373.s001.docx]

Supporting information:

Table S1 Reference sequences downloaded from GenBank. Columns in gray indicated possible misidentifications.

| Genebank accession No. | Species name | Voucher | Reference |
| --- | --- | --- | --- |
| FJ518943.1 | *Carcharodon carcharias* | yes | Wong *et al*.(2009) |
| DQ108328.1 | *Carcharodon carcharias* | yes | Ward *et al*.(2005) |
| FJ519083.1 | *Carcharhinus falciformis* | yes | Wong *et al*. (2009) |
| FJ519084.1 | *Carcharhinus falciformis* | yes | Wong *et al*. (2009) |
| EU398638.1 | *Carcharhinus plumbeus* | yes | Ward *et al*.(2008) |
| EU398639.1 | *Carcharhinus plumbeus* | yes | Ward *et al*.(2008) |
| JQ654711.1 | *Carcharhinus plumbeus* | no | Little *et al.* (2012) |
| FJ519001.1 | *Carcharhinus leucas* | yes | Wong *et al*.(2009) |
| EF609311.1 | *Carcharhinus leucas* | yes | Ward and Holmes (2007) |
| JQ693102.1 | *Scoliodon macrorhynchos* | no | Chen *et al.* (Direct submission) |
| EF539321.1 | *Squalus montalbani* | yes | Ward *et al.*(2007) |
| EU398513.1 | *Alopias pelagicus* | yes | Ward *et al*.(2008) |
| EU398516.1 | *Alopias pelagicus* | yes | Ward *et al*.(2008) |
| EU398583.1 | *Carcharhinus albimarginatus* | yes | Ward *et al*.(2008) |
| EU398586.1 | *Carcharhinus albimarginatus* | yes | Ward *et al*.(2008) |
| EU398602.1 | *Carcharhinus brevipinna* | yes | Ward *et al*.(2008) |
| EU398603.1 | *Carcharhinus brevipinna* | yes | Ward *et al*.(2008) |
| EU398890.1 | *Isurus oxyrinchus* | yes | Ward *et al*.(2008) |
| EU398892.1 | *Isurus oxyrinchus* | yes | Ward *et al*.(2008) |
| EU398896.1 | *Isurus oxyrinchus* | yes | Ward *et al*.(2008) |
| EU399018.1 | *Sphyrna zygaena* | yes | Ward *et al*.(2008) |
| FJ519057.1 | *Carcharhinus brachyurus* | yes | Wong *et al.* (2009) |
| FJ519059.1 | *Carcharhinus brachyurus* | yes | Wong *et al*. (2009) |
| FJ519531.1 | *Sphyrna zygaena* | yes | Wong *et al.* (2009) |
| JN315443.1 | *Sphyrna lewini* | no | Caballero *et al*. (2012) |
| JQ654702.1 | *Carcharodon carcharias* | no | Little *et al.* (2012) |
| JQ654704.1 | *Lamna nasus* | no | Little *et al.* (2012) |
| JQ654705.1 | *Isurus oxyrinchus* | no | Little *et al.* (2012) |
| JQ654709.1 | *Carcharhinus longimanus* | no | Little *et al.* (2012) |
| EU398627.1 | *Carcharhinus longimanus* | yes | Ward *et al*.(2008) |
| JQ654710.1 | *Carcharhinus limbatus* | no | Little *et al.* (2012) |
| JQ654710.1 | *Carcharhinus limbatus* | no | Little *et al.* (2012) |
| JN989310.1 | *Carcharhinus limbatus* | yes | Moore *et al*. (Unpublished) |
| JQ654713.1 | *Prionace glauca* | no | Little *et al.* (2012) |
| EU869837.1 | *Prionace glauca* | yes | Ward *et al.* (2008) |
| JQ654714.1 | *Carcharhinus galapagensis* | no | Little *et al.* (2012) |
| JQ654714.1 | *Carcharhinus galapagensis* | no | Little *et al.* (2012) |
| FJ519094.1 | *Carcharhinus galapagensis* | yes | Wong *et al*. (2009) |
| JQ654715.1 | *Galeocerdo cuvier* | no | Little *et al.* (2012) |
| JQ654715.1 | *Galeocerdo cuvier* | no | Little *et al.* (2012) |
| HQ171675.1 | *Galeocerdo cuvier* | yes | Doukakis *et al.*(2011) |
| DQ108224.1 | *Deania calcea* | yes | Ward et al.(2005) |
| DQ108329.1 | *Alopias superciliosus* | yes | Ward *et al*.(2005) |
| DQ108330.1 | *Alopias superciliosus* | yes | Ward *et al*.(2005) |
