# Supplementary material for: DNA Barcoding of Shark Meats Identify Species Composition and CITES-Listed Species from the Markets in Taiwan
Source: PLoS One. 2013 Nov 18;8(11):e79373. doi: 10.1371/journal.pone.0079373 (PMC3832526; doi:10.1371/journal.pone.0079373)
Supplement: Table S2 — Results of genetic barcoding and the species composition in different sampling regions. (DOCX) [file pone.0079373.s002.docx]

Table S2 Results of genetic barcoding and the species composition in different sampling regions.

| **Samples** | **Taipei City** | **New Taipei City** | **Taoyuan County** | **Hsinchu County** | **Miaoli County** | **Nantou County** | **Taichung County** | **Changhua County** |
| --- | --- | --- | --- | --- | --- | --- | --- | --- |
| ***Alopias pelagicus*** | **3** |  |  |  |  |  |  |  |
| ***Alopias superciliosus*** |  |  |  |  |  |  |  |  |
| ***Carcharhinus albimarginatus*** | **2** |  |  |  |  |  |  |  |
| ***Carcharhinus plumbeus*** |  |  |  |  |  |  |  |  |
| ***Carcharhinus brachyurus*** |  |  |  |  |  |  |  |  |
| ***Carcharhinus brevipinna*** |  |  |  |  |  |  |  |  |
| ***Carcharhinus falciformis*** | **6** |  | **1** | **3** | **3** |  |  |  |
| ***Carcharhinus galapagensis*** | **1** |  |  |  |  |  |  |  |
| ***Carcharhinus leucas*** |  |  |  |  |  |  |  |  |
| ***Carcharhinus limbatus*** |  |  |  |  |  |  |  |  |
| ***Carcharhinus longimanus*** |  |  |  |  |  |  |  |  |
| ***Carcharodon carcharas*** |  |  |  |  |  |  |  |  |
| ***Galeocerdo cuvier*** |  |  |  |  |  |  |  |  |
| ***Isurus oxyrinchus*** | **14** | **1** | **8** | **3** | **1** | **2** | **1** |  |
| ***Prionace glauca*** | **6** |  |  | **1** |  |  |  |  |
| ***Scoliodon laticaudus*** |  |  |  | **1** |  |  |  | **1** |
| ***Sphyrna lewini*** |  |  |  |  | **2** |  |  |  |
| ***Sphyrna zygaena*** |  |  | **1** |  |  |  |  |  |
| ***Deania sp.*** |  |  |  |  |  |  |  |  |
| ***Squalus montalbani*** |  |  |  |  |  |  |  |  |
| **Total** | **32** | **1** | **10** | **8** | **6** | **2** | **1** | **1** |

| **Yunlin County** | **Chiayi City** | **Chiayi County** | **Kaohsiung City** | **Pingtung City** | **Pingtung County** | **Taitung County** | **Ilan County** |  |
| --- | --- | --- | --- | --- | --- | --- | --- | --- |
| 2 |  |  | 1 | 11 | 52 | 53 | 3 | 125 |
|  |  |  | 1 | 2 | 1 | 28 | 7 | 39 |
| 1 |  |  |  |  |  | 6 |  | 9 |
|  |  |  |  |  |  | 1 |  | 1 |
|  |  |  |  |  |  |  | 1 | 1 |
|  |  |  |  | 1 |  | 2 | 2 | 5 |
| 1 | 1 | 1 | 2 | 1 | 46 | 60 |  | 125 |
|  |  |  | 1 |  |  | 2 | 1 | 5 |
|  |  |  |  |  |  | 2 |  | 2 |
|  |  |  |  |  |  | 3 | 1 | 4 |
|  |  |  |  |  | 6 | 3 |  | 9 |
|  |  |  |  |  |  | 1 |  | 1 |
|  |  |  | 1 |  |  | 7 |  | 8 |
|  |  |  | 1 |  | 7 | 53 | 1 | 92 |
| 1 |  |  | 1 | 1 |  | 80 | 8 | 98 |
|  |  |  |  |  |  |  |  | 2 |
|  |  |  |  |  |  | 8 | 4 | 14 |
|  |  |  |  |  | 1 | 3 |  | 5 |
|  |  |  |  |  |  | 1 |  | 1 |
|  |  |  |  |  | 2 |  |  | 2 |
| 5 | 1 | 1 | 8 | 16 | 115 | 313 | 28 | 548 |
